# Image intensification - A solution for difficult guidewire insertion for central venous access: A case report

**DOI:** 10.1016/j.amsu.2019.12.005

**Published:** 2020-01-03

**Authors:** Laurence Weinberg, Matthew Yii BBiomed, Michael Li BBiomed, Maleck Louis BBiomed, Dong Kyu Lee, Laurie Doolan

**Affiliations:** a Department of Anesthesia, Austin Health, 145 Studley Road, Heidelberg, 3084, Australia; b Dept. of Anesthesiology and Pain Medicine, Korea University Guro Hospital, Guro-Gu, Seoul, 08308, Republic of Korea

**Keywords:** Central venous catheter, Image intensification, Ultrasound, Guidewire, Cardiac surgery, Anaesthesia

## Abstract

**Presentation of case:**

A 56-year old male presented for an elective redo-sternotomy, aortic valve replacement, tricuspid valve annuloplasty, and coronary artery bypass grafting. During central vascular access using a standard Seldinger technique, resistance to two spring-wire guide wires was encountered when the wires were advanced through the patient's internal jugular vein. Ultrasound provided limited views of the anatomical path of the guidewires and was unable to provide visualisation of the level or cause of obstruction. We describe the application of continuous image intensification to successfully identify the anatomical location and safe circumnavigation of guidewire obstruction during the insertion of a central venous and pulmonary artery catheter for cardiac surgery.

**Discussion:**

The use of x-ray image intensification enabled the immediate identification of the cause of obstruction, minimising further attempts at guidewire insertion and subsequent complications. The direct real-time visualisation allowed for manoeuvres such as wire manipulation, rotation and advancement to be safely performed.

**Conclusion:**

Image intensification may decrease malposition rates and mechanical complications associated with difficult central venous catheterisation. Further research comparing the safety and efficiency of ultrasound-guided and fluoroscopy-guided CVC insertion should be contemplated.

## Highlights

• Multiple attempts at central line placement are associated with higher rates of complications.
• Image intensification can be used with ultrasound to facilitate the safe placement of central venous/pulmonary artery catheters.
• Image intensification can provide continuous and real-time visualisation of the guidewire along the vessel's anatomical course.
• Image intensification may decrease malposition and mechanical complications associated with difficult central venous access.

## Abbreviations

IJV: internal jugular vein
US: ultrasound
CVC: central venous catheter
PAC: pulmonary artery catheter

## 1 Introduction

The Seldinger technique performed under ultrasound guidance is the preferred method for obtaining internal jugular vein (IJV) vascular access for high risk cardiac surgery. Insertion of an IJV catheter is conventional care during cardiac surgery to facilitate invasive monitoring, infusion of vasoactive medications and inotropes, and measurement of cardiac function. However, there are numerous risks associated with the insertion of invasive lines, and complications from guidewire insertion are well reported [[1], [2], [3], [4]]. Published data suggests that the use of ultrasound (US) for vascular access reduces guidewire complication rates when compared to a “blind” technique using clinical landmarks only [[4], [5], [6], [7]].

Accordingly, use of US is the standard of care in clinical practice for vascular access techniques such as central venous (CVC) and pulmonary artery catheters (PAC) for cardiac surgery. We present a case where US was limited in its ability to diagnose the obstruction of two guidewires that were inserted to facilitate the insertion of a CVC and PAC. In this case report, we describe the application of continuous x-ray image intensification to successfully identify the anatomical location of guidewire obstruction and facilitate the safe advancement of guidewires through the IJV to the level of the right IJV and caval-atrial junction. This case report complies with the Surgical Case Report (SCARE) guidelines [8].

## 2 Presentation of case

A 56-year old male (157 cm tall, 75.6 kg, body mass index 30.7 kg/m^2^) presented for an elective redo-sternotomy, aortic valve replacement, tricuspid valve annuloplasty, and coronary artery bypass grafting. Symptoms included New York Heart Association class III dyspnoea, unstable angina and worsening lethargy. Past medical history included ischemic heart disease requiring previous coronary artery bypass grafts, aortic stenosis, tricuspid regurgitation, atrial fibrillation, and a dual chamber permanent pacemaker for sick sinus syndrome. Other patient cardiovascular risk factors included controlled hypertension, type 2 diabetes mellitus, obstructive sleep apnoea, obesity and dyslipidemia. His Euroscore II was 20.9%.

### 2.1 Investigations

Preoperative transthoracic echocardiography revealed preserved left ventricular function, severe bi-atrial enlargement, a dilated right ventricle with moderate systolic impairment, and a severely calcified bicuspid aortic valve with severe aortic stenosis. Medications included twice daily metformin (1 g), and daily aspirin (150 mg), bisoprolol (5 mg), digoxin (250 μg), frusemide (40 mg), atorvastatin (40 mg), perindopril (4 mg), nicorandil (10 mg) and rivaroxaban (20 mg).

**Fig. 1:**
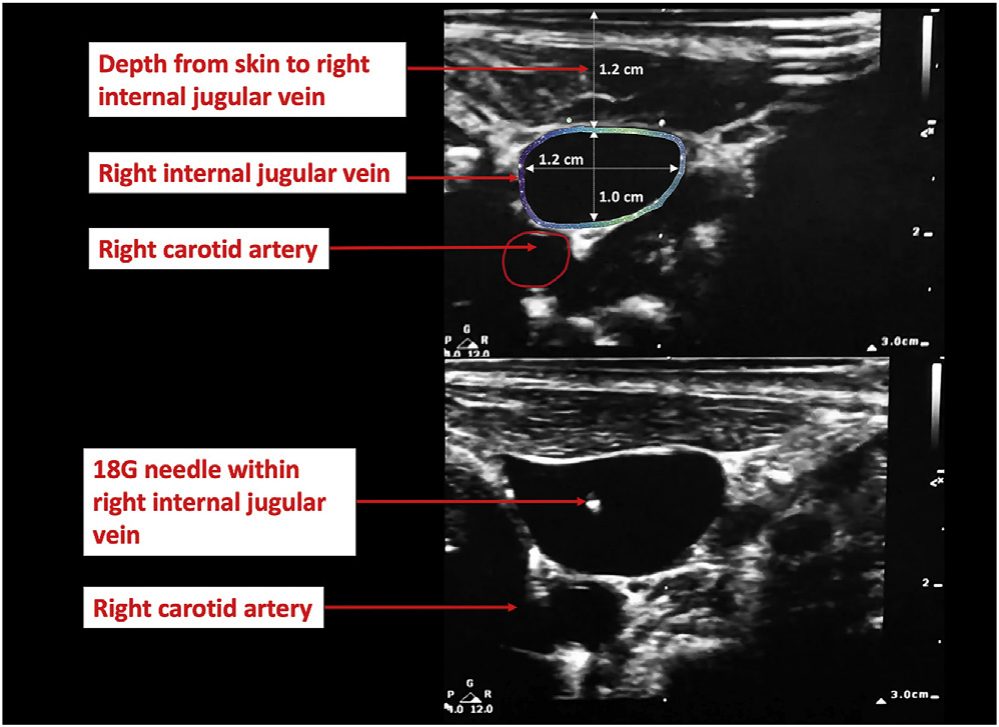
Ultrasound demonstrating the anatomical vascular structures of the right neck with the introducer needle successfully entering right internal jugular vein.

On arrival to the operating room a peripheral intravenous cannula and an arterial monitoring line were inserted by the anaesthesiologist. The patient was placed supine in the Trendelenberg position. Ultrasound of the neck revealed a patent 12 mm right IJV, with absence of thrombus and no evidence of stenosis. The IJV was anterior and lateral to the carotid artery confirming a normal anatomical location. The pacemaker leads were not visible in the vein (Fig. 1).

Using a strict aseptic technique, the anesthesiologist inserted a multi-lumen central venous catheter (Arrow International Inc, Reading PA) into the right IJV using the Seldinger technique. Using dynamic real-time US guidance an introducer needle attached to a 5 mL Luer slip syringe was introduced through the skin. Negative pressure was applied by pulling back on the plunger of the syringe and the needle was advanced directly into the right IJV (Fig. 1). When blood was aspirated, the needle was stabilised, and the syringe removed. A spring-wire guide (0.81 mm diameter x 45 cm; straight soft tip on one end - “J" tip on other) was inserted through the hub of the needle, "J" tip first, and advanced 10 cm into the vein. At this point, a firm resistance to wire insertion was encountered. Short and long axis US views confirmed the wire to be in the IJV; however, provided limited views of the anatomical path of the guidewire and were unable to provide adequate visualisation of the level or cause of obstruction.

### 2.2 Differential diagnosis

Differential diagnoses considered were a kink of the guidewire obstructing its path, external compression of the IJV from an inadvertent haematoma, wire malposition (vessel perforation with the guidewire in an extravascular location) or a mechanical obstruction caused by the pacemaker leads, intravascular stenosis or thrombus, or other anatomical structure (e.g. valve). The wire was able to be easily withdrawn proximally within the vessel but would consistently not advance beyond the 10 cm position. A decision was made to temporarily leave the wire in situ and proceed with the insertion of a second spring-wire guide (0.89 mm diameter. x 45 cm, straight soft tip on one end - "J" tip on other). Firm resistance to wire insertion was again encountered at a depth of 10 cm. Short and long axis US views confirmed that the second wire was visible in the IJV, but were unable to provide visualisation of the level or cause of obstruction.

### 2.3 Treatment

**Fig. 2:**
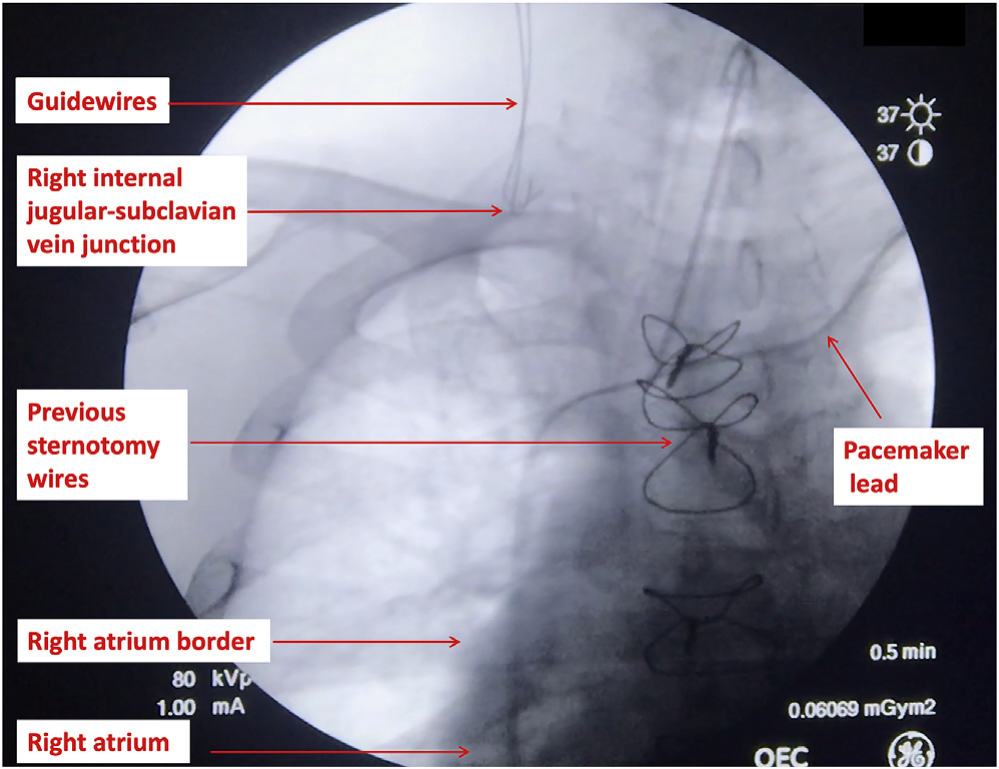
Image intensification demonstrating obstruction of the guidewires at the junction of the right internal jugular-subclavian vein.

**Fig. 3:**
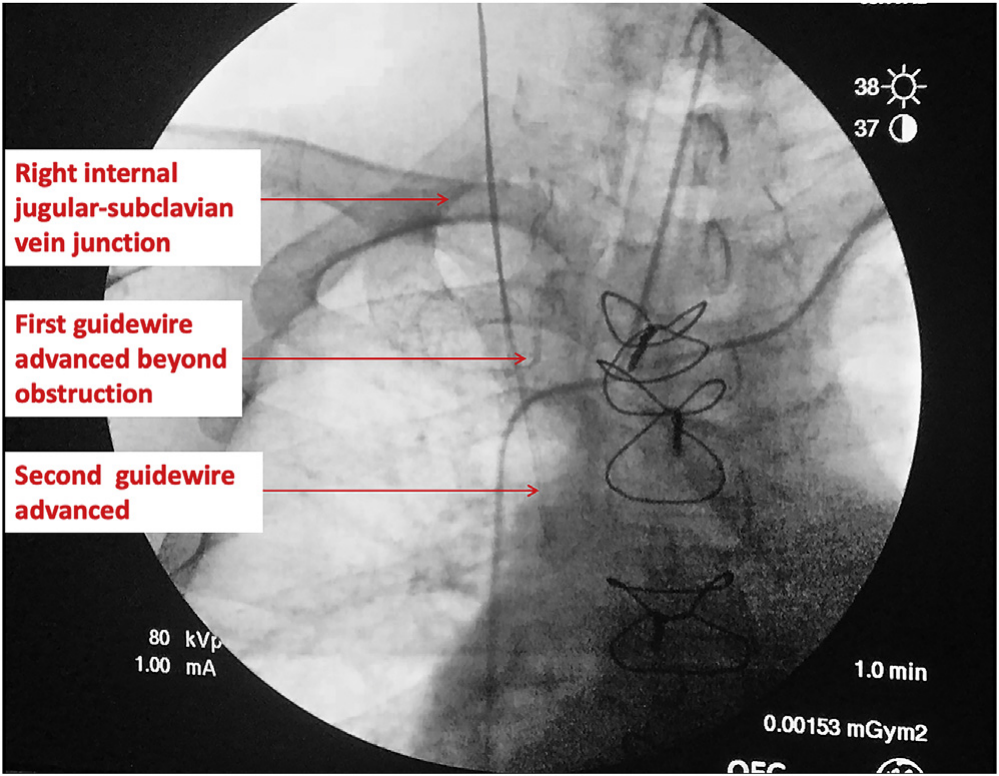
Image intensification demonstrating distal tip of two guidewires positioned at the caval-atrial junction.

Using continuous x-ray image intensification, the cause of guidewire obstruction appeared to be at the level of the right internal jugular-subclavian vein juncture, with both the "J" tip of each wire curling backwards (Fig. 2). A 3 mL intravenous injection of radiocontrast iodine (Visipaque 320 mg I/mL, GE Healthcare, NSW, Australia) was injected into the IJV and demonstrated no intravascular stenosis or thrombus. Each wire was cautiously withdrawn by 1–2 cm, rotated and re-orientated, suggesting that the cause of the obstruction was likely a valve at the level of the subclavian vein-superior vena cava junction. The real-time observation of guidewire manoeuvring enabled re-advancement of the guidewire freely beyond the obstruction (Fig. 3). The distal ends of both wires were then positioned at the level of the caval-atrial junction (Video 1). The total dose of radiation used was 2.9 mSv [2]. The patient tolerated the procedure well, and surgery proceeded uneventfully. There were no postoperative complications and the patient was discharged home on postoperative day 7.

Supplementary video related to this article can be found at https://doi.org/10.1016/j.amsu.2019.12.005.

The following is/are the supplementary data related to this article:

Video 1Image intensification demonstrating identification of obstruction and manipulation of guidewire past site of resistance.

## 3 Discussion

We report the use of image intensification for difficult guidewire insertion for central venous access for high-risk cardiac surgery. The use of central venous and pulmonary artery catheters in patients undergoing redo cardiothoracic surgery is common. Whilst ultrasound and fluoroscopy are frequently used by radiologists to guide placement of central venous ports via the IJV [10], the use of fluoroscopy by anaesthesiologists or critical care clinicians to facilitate CVC placement is uncommon. Although the Seldinger technique combined with US is established as standard of care for the safe insertion of central venous catheters in many institutions [11], complications of vascular access are well-recognised [4,12,13]. A recent meta-analysis reported the prevalence of CVC malposition to be up to 6.8% [14] and the prevalence of iatrogenic pneumothorax between 0.1 and 3.3% [15]. The incidence of small needle arterial puncture during vascular access techniques is reported to be approximately 4.2–9.3% [16,17].

Whilst uncommon and frequently unrecognised, the reported incidence of arterial puncture with subsequent dilation or catheterisation of the artery is 0.1–1.0% [16,17]. This more serious complication can result in pseudoaneurysm, haemorrhage, and stroke. In addition, complications from central venous catheter insertion have been associated with patient mortality [2,18]. The exact rate of these complications varies depending on patient factors (morbid obesity, anatomical variation), proceduralist factors (level of experience, use and proficiency with vascular US), and technical factors (insertion site, needle and catheter size, number of insertion attempts) [19,20].

The use of vascular US minimises the risk of complications by facilitating the position and patency of the vascular structure, guiding needle puncture and confirming the needle, wire and catheter position within the vessel [21,22]. Plain radiographs post-procedure are also commonly used for confirmation of catheter placement and to detect complications including pneumothorax and catheter misposition [23,24]. The use of x-ray image intensification for the routine insertion of CVC and PAC placement is not commonly reported. In a study by Weinberg et al. [9] video fluoroscopy was reported to be a practical method to guide PAC flotation in cardiac surgery patients who are at a higher risk for PAC related complications. The authors reported a significant reduction in the time taken to correctly position the catheter, a significant reduction in the median number of attempts, and a significant reduction in the composite complication rate for malposition and arrhythmias (16% vs 52%) when compared to the traditional pressure waveform flotation technique [9]. Practical limitations of the use of image intensification include its inconvenience, time use, and need for dedicated radiographer. A case series by Gautam et al. also reported that forced saline flushes under the guidance of image intensification was effective at repositioning and straightening misplaced central venous catheters [25]. Whilst use of image intensification exposes the patient and staff to radiation, in the present case, a radiation dose 2.9 mSv [2] was used, equivalent to less than one year's exposure to normal sunlight.

In conclusion, image intensification can be used to facilitate safe vascular access placement in high-risk cardiac patients by providing continuous and real-time visualisation of the guidewire. This in turn can minimise multiple attempts at guidewire insertion by providing immediate visualisation of the guidewire along the vessel's anatomical pathway, and allowing for manoeuvres such as wire manipulation, rotation and advancement to be safely performed when appropriate. We postulate that image intensification used in conjunction with US may decrease malposition rates and mechanical complications associated with difficult central venous catheterisations in high-risk cardiac surgical patients. This report allows the generation of hypotheses and accumulation of scientific data about use of image intensification amongst non-radiologists. Further research comparing the safety and efficiency of ultrasound-guided and fluoroscopy-guided CVC insertion should be contemplated.

## Ethical approval

This is not a clinical trial. Informant written participant consent has been obtained in compliance with Austin Health Research Ethics Committee requirements.

## Sources of funding

None.

## Consent

Written participant consent has been obtained in compliance with Austin Health Research Ethics Committee requirements.

## Author contribution

Laurence Weinberg, Laurie Doolan: principal anaesthesiologist, case report initiation and planning, patient consent, collation of all images, and writing of report. Matthew Yii, Dong Kyu Lee, Michael Li, Maleck Louis: literature review, preparation of images, writing of manuscript.

## Registration of research studies

1. Name of the registry: not applicable
2. Unique Identifying number or registration ID: not applicable
3. Hyperlink to the registration (must be publicly accessible): not applicable

## Guarantor

A/Prof Laurence Weinberg, Director of Anaesthesia, Austin Hospital.

145 Studley Road, Heidelberg, Victoria, 3084, Australia.

laurence.weinberg@austin.org.au

Phone: +613 9496 5000.

## Declarations of interest

None.

## Provenance and peer review

Not commissioned, externally peer reviewed.

## Declaration of competing interest

The authors confirm that they have no competing interests to declare.
